# Integration of ChatGPT Into a Course for Medical Students: Explorative Study on Teaching Scenarios, Students’ Perception, and Applications

**DOI:** 10.2196/50545

**Published:** 2024-08-22

**Authors:** Anita V Thomae, Claudia M Witt, Jürgen Barth

**Affiliations:** 1 Institute for Complementary and Integrative Medicine, University Hospital Zurich and University of Zurich, Zurich, Switzerland; 2 Institute of Social Medicine, Epidemiology and Health Economics, Charité – Universitätsmedizin Berlin, Corporate member of Freie Universität Berlin, Humboldt-Universität zu Berlin, and Berlin Institute of Health, Berlin, Germany

**Keywords:** medical education, ChatGPT, artificial intelligence, information for patients, critical appraisal, evaluation, blended learning, AI, digital skills, teaching

## Abstract

**Background:**

Text-generating artificial intelligence (AI) such as ChatGPT offers many opportunities and challenges in medical education. Acquiring practical skills necessary for using AI in a clinical context is crucial, especially for medical education.

**Objective:**

This explorative study aimed to investigate the feasibility of integrating ChatGPT into teaching units and to evaluate the course and the importance of AI-related competencies for medical students. Since a possible application of ChatGPT in the medical field could be the generation of information for patients, we further investigated how such information is perceived by students in terms of persuasiveness and quality.

**Methods:**

ChatGPT was integrated into 3 different teaching units of a blended learning course for medical students. Using a mixed methods approach, quantitative and qualitative data were collected. As baseline data, we assessed students’ characteristics, including their openness to digital innovation. The students evaluated the integration of ChatGPT into the course and shared their thoughts regarding the future of text-generating AI in medical education. The course was evaluated based on the Kirkpatrick Model, with satisfaction, learning progress, and applicable knowledge considered as key assessment levels. In ChatGPT-integrating teaching units, students evaluated videos featuring information for patients regarding their persuasiveness on treatment expectations in a self-experience experiment and critically reviewed information for patients written using ChatGPT 3.5 based on different prompts.

**Results:**

A total of 52 medical students participated in the study. The comprehensive evaluation of the course revealed elevated levels of satisfaction, learning progress, and applicability specifically in relation to the ChatGPT-integrating teaching units. Furthermore, all evaluation levels demonstrated an association with each other. Higher openness to digital innovation was associated with higher satisfaction and, to a lesser extent, with higher applicability. AI-related competencies in other courses of the medical curriculum were perceived as highly important by medical students. Qualitative analysis highlighted potential use cases of ChatGPT in teaching and learning. In ChatGPT-integrating teaching units, students rated information for patients generated using a basic ChatGPT prompt as “moderate” in terms of comprehensibility, patient safety, and the correct application of communication rules taught during the course. The students’ ratings were considerably improved using an extended prompt. The same text, however, showed the smallest increase in treatment expectations when compared with information provided by humans (patient, clinician, and expert) via videos.

**Conclusions:**

This study offers valuable insights into integrating the development of AI competencies into a blended learning course. Integration of ChatGPT enhanced learning experiences for medical students.

## Introduction

Since its public launch in November 2022, ChatGPT (OpenAI), as a text-generating artificial intelligence (AI), has garnered significant attention in academic education overall and particularly in the field of medical education. Besides endeavors such as exams in the field of medicine [1,2], there are many other opportunities to implement ChatGPT in medical education [3,4]. However, these opportunities also have certain challenges such as overreliance, plagiarism, and privacy concerns [5]. Previous research has suggested the need for the advancement of knowledge, interpretation, and application of AI in the context of medical education [6], thereby underscoring the importance of acquiring practical skills essential for using AI in one’s future professional career. The lack of integrating AI into medical education has been described [7,8]. Up to now, there are, however, few specific proposals on how to implement text-generating AI into existing courses. Recent studies exemplify the integration of ChatGPT primarily as a tool for training communication skills among medical students [9,10] or as a supporting tool in problem-based learning scenarios [11].

In our elective course, “Placebo and Nocebo,” which was offered to medical students at the University of Zurich, we integrated content generated with ChatGPT into various teaching units by using different learning scenarios. The overall aim of this course was to teach medical students concepts related to the topic of placebo and nocebo.

Within the course, the importance of expectations regarding medical treatment, as raised by specific information and the corresponding impacts on treatment outcomes, were presented. Methods for phrasing information for patients concerning medical treatment, including benefits and potential side effects, was another key topic of this course. One possible application of ChatGPT in the clinical context could be to support the writing of information for patients in order to educate or prepare patients for upcoming treatments [12,13]. Such information must be clear and safe. Clarity includes readability and understandability of the presented information [14]. With regard to safety, information should present concerning potential side effects in a correct but layperson-friendly way, and the positive framing of side effects is encouraged [15,16].

In this explorative study, we wanted to investigate how medical students evaluate the integration of ChatGPT teaching units into the course and their perceived importance of text-generating AI–related competencies during their studies. Furthermore, we wanted to explore how personal characteristics such as sex and openness to digital innovation are related to these outcomes. By using information for patients written using ChatGPT, we wanted to further explore how medical students in this course assess the use of ChatGPT-created content as a source of information for patients and its respective persuasiveness.

## Methods

### Procedure

Medical students (third bachelor and first master) were invited to enrol themselves in the elective course “Placebo and Nocebo” at the University of Zurich in spring 2023. The course comprised 28 teaching units (45 min each), 3 of which integrated ChatGPT. The course was set up as a blended learning course combining 13 teaching units delivered as e-learning and 15 teaching units delivered as in-person lectures. The course description was available to students before enrollment and indicated that the course would include a scientific evaluation and that the results would be published. All interaction with ChatGPT was performed on a personalized teacher’s account (AT) rather than by students due to data privacy issues.

**Figure 1.**
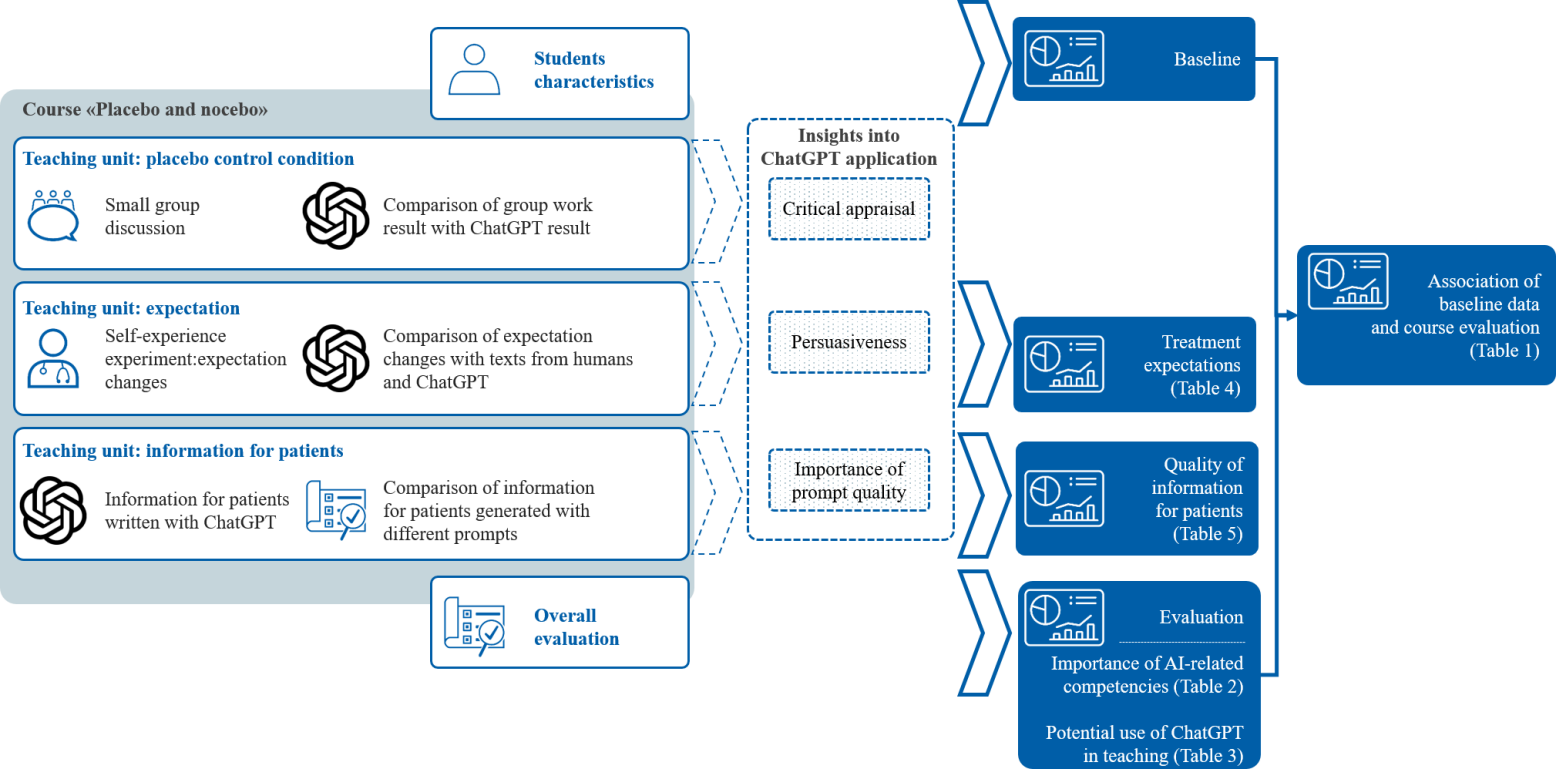
Integration of ChatGPT into different teaching units during the “Placebo and Nocebo” course taught at the University of Zurich.

The teaching units of the course and the corresponding insights gained into ChatGPT application as well as the data gathered are summarized in Figure 1.

Baseline and additional data, along with the overall course evaluation data, were collected via web both before and after the course.

In the teaching unit “Placebo control condition,” the students were divided into working groups. They developed a placebo control condition for a clinical trial using a checklist in a problem-based learning scenario. Subsequently, the students presented their results in the plenary. These results were compared with suggestions from ChatGPT and discussed in the plenary, emphasizing a critical appraisal of the ChatGPT-generated solution.

In the teaching unit “Expectations,” the students participated in an experiment. Through web-based questionnaires, they were asked about their individual outcome expectations regarding an acupuncture treatment for headaches. This specific symptom and treatment were selected due to the likelihood that students may have familiarity with both. We created 4 different videos that were 60 to 80 seconds in length, which were presented sequentially over 4 weeks (1 video per week). The videos were animated voice-overs. Each video delivered supportive information regarding the treatment of acute headaches using acupuncture, but different protagonists and different information were used (expert opinion, clinician opinion, patient experience, and general information from the internet). For example, the expert opinion focused on the evidence, whereas the patient reported on her own experience. The video transcripts in English are presented in Multimedia Appendix 1. Directly before and after each video, the medical students answered 5 questions regarding their own expectations concerning acupuncture within the e-learning of the course. This pre-post-measurement provided an opportunity to assess the extent to which each video impacted the expectations of the students. The persuasiveness of text written using ChatGPT was compared with text provided by human beings. The information written using ChatGPT 3.5 was labeled as “general information from the internet.” The same text was used in the subsequent teaching unit “Information for patients” using the extended prompt from that unit.

Before the teaching unit “Information for patients,” students learned and practiced the rules for developing content intended for patients, along with the criteria used to review such information [15,16]. During the lecture, students were divided into 9 groups containing 4 to 6 students each. They applied these criteria within their groups to review 2 different pieces of information for patients written with ChatGPT 3.5 using digital whiteboards. Aiming to provide insight into the importance of prompt quality, the first information for patients was written with a rather basic prompt (“Can you please write a patient information about the effectiveness of acupuncture for tension headaches. The text should be no longer than 180 words.”), whereas the second extended prompt included the review criteria (“Can you please change this text so that it is generally understandable for patients? Please formulate statements about effects and side effects in the sense of positive framing. Also make sure that patient safety is guaranteed. The text should be no longer than 180 words”). The students also marked content in each of the descriptions which they found “problematic” within the information for patients (eg, terminology). The review decisions and the rationale behind each group’s choices were discussed in the plenary. The prompts and corresponding answers written with ChatGPT 3.5 as well as the content labeled as “problematic” by the groups are shown in Multimedia Appendix 1.

### Outcomes

#### Treatment Expectations

During the experiment in the teaching unit “Expectation,” students’ expectations concerning acupuncture treatment for headaches were assessed using the Expectation for Treatment Scale [17] before and after the students watched the information provided in the video. The Expectation for Treatment Scale contains 5 items assessing the individual expectations regarding the effectiveness of a specific treatment. Total scores could range from 5 to 20, with higher values indicating higher expectations concerning acupuncture treatment.

#### Quality of Information for Patients

During the teaching unit “patient information,” in 9 subgroups, students reviewed the quality of information for patients written with ChatGPT in terms of three criteria: (1) comprehensibility (lay language), (2) patient safety, and (3) correct application of communication rules taught in the course (positive framing). The categories for these judgments were “Fully met,” “Partly met,” and “Not met.” Furthermore, the medical students were instructed to mark unclear, ambiguous, and critical content in the information for patients.

#### Evaluation

The integration of ChatGPT into different teaching units of the course was evaluated based on the Kirkpatrick Model [18,19], encompassing 3 levels: satisfaction, learning progress, and application. Ratings were assigned on a scale ranging from 1 (no agreement) to 6 (full agreement). We built aggregated scores for satisfaction (Cronbach *α*=.90), learning progress (Cronbach *α*=.80), and application (Cronbach *α*=.72) with good to excellent internal consistency based on 4 items per level. The results regarding single items are reported in Multimedia Appendix 1.

### Additional Data

Baseline information of the students (age, sex, and previous experience with text-generating AI) was collected anonymously via the web. Openness to digital innovation was assessed using 4 items that have a similar phrasing to that used in the NeoFFI (NEO Five Factors Inventory) for assessing openness to experience [20]. Each item was assessed on a scale ranging from 1 (no agreement) to 6 (full agreement). The aggregated score ranged from 1 to 6, and the four items had excellent internal validity (Cronbach *α*=.95). Furthermore, medical students were asked about the potential of AI in medical education and medicine in general. The students evaluated the importance of 5 AI competencies in medical education on a scale ranging from 1 (no agreement) to 6 (full agreement). Competencies were selected based on the proposal of Caliskan et al [21]. Additionally, students shared their thoughts concerning the potential use of text-generating AI in medical education in response to an open question.

### Data Analysis

Baseline data are summarized as median and IQR. Data from the teaching unit “Expectations,” were analyzed using paired *t* tests to explore pre-post differences in expectations. The magnitude of the effect is expressed as mean difference with the respective 95% CI and as effect size. Data drawn from the teaching unit “Information for patients,” are described as counts on group level. Data drawn from the overall evaluation are presented as median and IQR for the 3 evaluation levels due to the skewed data distribution. Spearman correlations between openness and the 3 evaluation levels were calculated for the total group and stratified by sex. The quantitative results concerning competencies are reported in a descriptive way. Quantitative analyses were conducted using IBM SPSS Statistics (Version 29). All analyses must be considered as exploratory.

We conducted a thematic analysis of the qualitative data using MAXQDA Software (release 20.4.2; Verbi) [22]. A team of 6 researchers created themes based on the answers provided by the students in 3 subgroups and coded the answers accordingly. The themes and codings associated with the subgroups were then harmonized through discussion, rearrangement, and intersubjective validation within the whole group.

### Ethical Considerations

We submitted the study synopsis to the Ethics Committee of Zurich, Switzerland, and, after review, they stated that the study did not fall under the regulations of the Human Research Act of Switzerland (BASEC-Nr. Req-2023‐00400).

## Results

### Study Participants

In total, 52 medical students (19 male and 33 female) participated in the “Placebo and Nocebo” course. The median age of the participants was 23 (IQR 22‐25) years. Of the 40 students who completed the overall evaluation, 43% (n=17) of participants reported having never or rarely used text-generating AI before. A third (14/40, 35%) of participants had used text-generating AI occasionally before, and 23% (n=9) participants reported frequent or very frequent previous use of text-generating AI.

### Overall Evaluation

Overall, the integration of ChatGPT into different teaching units of the course was evaluated very positively with high satisfaction scores (median 5.12, IQR 4.31‐5.75), high perceived learning progress (median 4.37, IQR 3.75‐5.25) due to the course and high applicability of the knowledge (median 4.75, IQR 4.25‐5.25). The results regarding single items are reported in Multimedia Appendix 1.

The 3 levels of satisfaction, progress, and applicability were correlated by approximately 0.50 to 0.60, thus indicating rather strong associations among all 3 learning levels (Table 1). More interestingly, the associations between the overall evaluation outcomes and participants’ sex and their openness to digital innovation are presented in the first 2 lines of Table 1. Sex was not associated with the evaluation outcomes of the course, but openness was strongly associated with satisfaction and to a lesser extent with applicability. No association between openness and progress was found.

Since the male sex was associated with higher openness, the associations for females and males were analyzed separately. There were no major differences between sex strata in the association of openness with satisfaction (female 0.500; male 0.652). However, the association of openness with progress (female: 0.150; male: 0.419) and applicability (female: 0.317; male: 0.783) differed between sexes. Male students with high openness also indicated higher learning progress and a higher applicability of the course content than female students.

**Table 1.** Associations of openness, sex, and the evaluation of the course (satisfaction, progress, and application).

		Sex	Openness	Satisfaction	Progress	Application
**Sex^a^**						
	Spearman correlation	1	0.462	0.037	−0.121	0.073
	*P* value	—^b^	.003	.82	.46	.65
**Openness**						
	Spearman correlation	0.462	^1^	0.483	0.119	0.399
	*P* value	.003	—	.002	.47	.01
**Satisfaction**						
	Spearman correlation	0.037	0.483	1	0.585	0.584
	*P* value	82	.002	—	<.001	<.001
**Progress**						
	Spearman correlation	−0.121	0.119	0.585	1	0.622
	*P* value	.46	.47	<.001	—	<.001
**Application**						
	Spearman correlation	0.073	0.399	0.584	0.622	1
	*P* value	.65	.01	<.001	<.001	—

a Female sex was coded as 1 and male sex as 2.

b Not applicable.

### Potential of AI in Medical Education and Medicine in General

As shown in Table 2, the perceived importance of AI-related competencies in other courses of the medical curriculum is high for the students. Among the suggested competencies, the assessment of the opportunities and limitations of text-generating AI received the highest rating, while competencies for basic understanding of how AI functions received the lowest rating but are still regarded as important.

Qualitative analysis of the open-ended question revealed areas in teaching and learning for which students see potential uses of ChatGPT. The themes identified and example quotations are shown in Table 3. Furthermore, students identified other potential uses of ChatGPT, namely, supporting clinical practice (eg, the use of ChatGPT in the context of documentation/patient reports, administrative work and support as a second opinion) and serving as a general information source. In addition, students made several statements regarding their opinions and values, such as the perceived lack of empathy in ChatGPT and the necessity for human supervision.

**Table 2.** Students’ perceived importance^a^ of artificial intelligence (AI)–related competencies in other courses.

AI-related competencies	Median (IQR)	
Competencies for assessing the opportunities and limitations of text-generating AI	5.0 (5.00‐6.00)	
Competencies for combining text-generating AI with professional knowledge	5.0 (4.00‐5.75)	
Competencies for assessing the value of text-generating AI in teaching, care and research	5.0 (4.00‐5.75)	
Competencies for the efficient and effective use of text-generating AI in patient care	5.0 (4.00‐6.00)	
Competencies pertaining to a basic understanding of how text-generating AI functions	4.0 (4.00‐5.00)	

a Each item was evaluated on a scale ranging from 1 (no agreement) to 6 (full agreement).

**Table 3.** Students’ ideas concerning the potential use of ChatGPT to support teaching and learning: Themes and quotations.

Themes	Quotations^a^ (representative examples)
General support for teaching and learning	*To generate good summaries of the learning material* (student 15) *Instead of emailing lecturers to clarify ambiguities regarding lecture content, ask ChatGPT* (student 37) *As exam preparation* (student 21)
ChatGPT as a form of writing support	*Maybe ChatGPT could be used to optimize one’s own texts* (student 13) *As a form of support, workload relief and the acceleration of processes* (student 38)
ChatGPT for patient case simulation	*ChatGPT could be used to simulate patients and thus practice what has been learned* (student 13)
Learning how to use ChatGPT	*We could learn in the context of these courses how we could use ChatGPT optimally for learning, for research* (student 13) *Assess the possibilities and limitations of such tools in a medical context* (student 22)

a These quotations were originally in German and were translated into English by the authors.

### Results Regarding Specific Teaching Units

#### Change in Treatment Expectations by Different Information

Expectations regarding treatment showed an increase after each of the 4 information videos was presented (Table 4). The strongest increase was observed for the video that shared patient experiences, whereas the video containing the ChatGPT content did not change expectations substantially. Information presented by a clinician or the expert opinion changed expectations moderately.

**Table 4.** Changes in treatment expectations by information presented as 4 videos (expert, clinician, patient, and ChatGPT).

	Expectation, mean difference (95% CI)		*t* test (*df*)	*P* value	Effect size
Expert (n=50)	0.649 (0.102‐1.178)		2.391 (49)	.021	0.338
Clinician (n=51)	0.941 (0.324‐1.558)		3.063 (50)	.004	0.429
Patient (n=51)	1.431 (0.746‐2.116)		4.198 (50)	<.001	0.588
ChatGPT (n=49)	0.449 (0.002‐0.896)		2.021 (48)	.049	0.289

#### Quality of Information for Patients

The students reviewed the information for patients written using ChatGPT using a basic prompt and judged the text most often as partially comprehensive, safe, and appropriate in terms of communication rules (Table 5). The students identified many terms and phrases that they deemed problematic for use in information for patients. Reasons mentioned by students in group discussion included, for instance, the use of too specific terminology with low readability and poor understandability (Multimedia Appendix 1). The information for patients written using ChatGPT using an extended prompt was reviewed very positively. Only a minority of the student groups indicated after the review that the criteria of comprehensibility, safety, and communication rules were only partly met or not met. The number of problematic terms identified by the students was much lower than the number of such terms in the first text.

**Table 5.** Students’ judgments (group decision counts) regarding the information for patients generated using ChatGPT using a basic or an extended prompt.

Review criteria	Basic prompt^a^			Extended prompt^b^		
	Fully met^c^	Partly met^c^	Not met^c^	Fully met^c^	Partly met^c^	Not met^c^
Comprehensibility	0	5	3	8	0	1
Safety	1	6	2	8	1	0
Communication rules	1	5	2	7	1	1

a Basic prompt: “Can you please write a patient information about the effectiveness of acupuncture for tension headaches? The text should be no longer than 180 words.”

b Extended prompt: “Can you please change this text so that it is generally understandable for patients? Please formulate statements about effects and side effects in the sense of positive framing. Also make sure that patient safety is guaranteed. The text should be no longer than 180 words.”

c Fully met: Rules are applied throughout the whole text; partly met: rules are sometimes applied, but not consistently throughout the whole text; not met: rules were not applied within the text.

## Discussion

### Principal Findings

Our study showed that integrating ChatGPT into medical courses is feasible, although the majority of the students had no or only limited experience using ChatGPT. The ChatGPT-enriched teaching units were highly appreciated by medical students, and this approach can be used as a stimulating teaching tool. Text generated using ChatGPT in a persuasion experiment (ie, information for patients to change treatment outcome expectations), a practical review exercise focusing on information for patients, and a problem-based learning scenario were suitable formats for our teaching units. All 3 formats used in this course were closely related to possible scenarios that may be relevant for medical students in their later professional careers. Medical students consider the acquisition of competencies related to text-generating AI to be highly important during their studies.

To support constructive learning for the students, the ChatGPT-enriched scenarios addressed different teaching strategies, namely, problem-solving, self-experience, and evaluation. The respective teaching units were embedded into the framework of the course rather than merely added, as also suggested by McCoy et al [23]. This goal was achieved by using ChatGPT directly to revisit or deepen teaching content drawn from other teaching units of the course. In general, the integration of ChatGPT in education demonstrated superiority in both evaluation results and knowledge outcomes. The findings from a medical communication course incorporating ChatGPT revealed positive student evaluations [24]. Direct comparisons of dental students’ knowledge after a ChatGPT-integrated teaching scenario with an AI-free scenario showed better learning progress [11]. Our course evaluation was based on the Kirkpatrick Model considering satisfaction, learning progress, and applicable knowledge as key assessment levels [18,19,25]. Based on the subjective assessment of the students, the evaluation results show that our approach can facilitate students’ understanding of the course content and allow them to explore the possibilities and limitations of text-generating AI.

Although nearly half of the students had no or only limited experience using text-generating AI, the evaluation was very positive. Hence, students’ previous experience with or interest in innovative technologies does not seem to be a necessary prerequisite for the introduction of such technologies into medical teaching. This confirms the findings of Weidener and Fischer from a survey of medical students across Germany, Austria, and Switzerland. In this study, less than half of the students had prior experience with ChatGPT or other AI-based chat applications but indicated a need for AI in medical education [7].

For the successful integration of AI into teaching modules, facilitating and impeding factors among students should be investigated. Openness to digital innovations might be an asset to facilitate learning with AI tools. It has been demonstrated in adults in the United States that users’ trust impacts both the intention to use and the actual use of ChatGPT [26]. Here, we showed that students with lower openness to digital innovation reported less satisfaction and lower applicability in our evaluations, which may be a result of their lower motivation to engage in teaching units with ChatGPT content. Consequently, less open students may also lack knowledge regarding the limitations of ChatGPT since they may avoid this technology in general. Sex is another factor that could lead to different receptions of AI-enriched courses. Higher use of AI tools in male persons has also been shown in students from different fields in Germany [27], possibly reflecting a higher openness to digital innovations. In our study, we also found similar associations of openness to digital innovations, progress, and applicability for male students. However, for female students, other factors beyond openness might affect progress and applicability.

Our evaluation results showed that the perceived importance of AI-related competencies for students is rated very high in general and covers a wide range of different competencies. The chosen competencies are similar to the categories of knowledge, interpretation, and application of AI that were revealed by teaching experts [6]. Some of these competencies were addressed in our teaching units. For example, the teaching unit “Information for patients,” illustrated the need to use a meaningful prompt and the importance of choosing relevant criteria when using ChatGPT to create information for patients. During the course, the quality of this information written using ChatGPT has been improved by incorporating important criteria for the text into the prompt. Students found information written using the extended prompt to be of higher quality, providing insights into the importance of prompt quality for the generated text. Several studies have investigated the application of ChatGPT and other large language models as tools for providing patient material, yielding promising results. According to Ayers et al [12], AI-generated text messages on health-related patient questions in a social media forum were superior to physician responses as rated by health care professionals. Tangadulrat et al [28] showed that both medical students and graduated doctors positively perceived using ChatGPT for creating patient educational materials. Patient material readability scores were considerably improved by the large language model as demonstrated by Rouhi et al [29]. Interestingly, the ChatGPT generated text using the extended prompt, was found to be the least persuasive within the expectation experiment. It did not change students’ expectations regarding a specific treatment substantially compared with information provided by humans (especially when compared with a patient statement). As the information written using ChatGPT was read by an artificial voice, while the other information was read by humans, the lower persuasiveness might be due to a lower acceptance of the artificial voice. A preference for human voices has been shown in other research [30].

Open-ended questions revealed misleading concepts, such as the use of text-generating AI to support patient documentation, a potential concern due to data protection issues as discussed by Eggmann et al [31]. Particularly, to circumvent problematic applications of text-generating AI in physicians’ later professional careers, the systematic integration of AI-related competencies into medical curricula is critical.

### Limitations

Our results lack generalizability with respect to the use of ChatGPT in other learning environments (eg, larger groups). Furthermore, the results cannot be generalized to the use of other generative AI (such as image-generating AI).

The evaluation items were based on the Kirkpatrick Model. However, all items were self-reported. Ideally, learning progress and application would be assessed with objective indicators, eg, based on progress tests and performance evaluations. Learning effects, especially at the level of application, would be larger if students used ChatGPT on their own, entering their own prompts rather than using answers written using ChatGPT based on teachers’ prompts. However, this scenario would cause problems with students’ data privacy and would be a course on its own.

Given the predefined structure and learning objectives of the course, it was unfortunately not possible to explore further the use of AI in generating information for patients and its respective change in expectation. Additionally, it would have been of advantage to reflect these questions not only with medical students but with patients as actual target groups of such information.

### Conclusions

According to the evaluation of medical students, integration of ChatGPT into an existing course is highly appreciated and enhances the learning experience. The development of AI-related competencies, including the phrasing of meaningful prompts during medical education, was perceived as very important by these medical students. The ability to critically appraise AI-generated information is also an important competency for medical students.

## Supplementary material

10.2196/50545 Multimedia Appendix 1 Detailed evaluation results and insight into teaching scenarios.

## Abbreviations

AI: artificial intelligence
NeoFFI: NEO Five Factors Inventory
