# Supplementary material for: Integration of ChatGPT Into a Course for Medical Students: Explorative Study on Teaching Scenarios, Students’ Perception, and Applications
Source: JMIR Med Educ. 2024 Aug 22;10:e50545. doi: 10.2196/50545 (PMC11360267; doi:10.2196/50545)
Supplement: Multimedia Appendix 1 [file mededu-v10-e50545-s001.pdf]

Multimedia Appendix

Integration of ChatGPT Into a Course for Medical Students: Insights From an Explorative Study Into Teaching Scenarios, Students Perception and Applications

Anita V. Thomae[1], Claudia M. Witt[1, 2], Jürgen Barth[1]

[1] Institute for Complementary and Integrative Medicine, University Hospital Zurich and University of Zurich, Zurich, Switzerland

[2] Charité – Universitätsmedizin Berlin, corporate member of Freie Universität Berlin, Humboldt-Universität zu Berlin, and Berlin Institute of Health, Institute of Social Medicine, Epidemiology and Health Economics, Berlin, Germany

Corresponding author:
Jürgen Barth, PhD
Institute for Complementary and Integrative Medicine, University Hospital Zurich and University of Zurich, Zurich, Switzerland
mail@juergen-barth.de

Table of Contents

Supplementary Material I: Overall evaluation 3
Single items 3
Supplementary Material II: Teaching unit “Expectation” 4
Questionnaire 4
Video content (transcript) 4
Supplementary Material III: Teaching unit “Information for patients” 6
Information for patients generated with ChatGPT 3.5 6
Basic prompt 6
Text generated with ChatGPT 6
Extended prompt 6
Text generated with ChatGPT 6
Screenshots from interactive whiteboard discussion 7
After basic prompt 7
After extended prompt 8

Supplementary Material I: Overall evaluation

Single items

	Median	IQR
**Openness to digital innovation**		
I often test new digital technologies.	4.0	3.00-5.00
I am very inquisitive about new digital technologies.	4.0	3.00-5.00
I enjoy testing new digital technologies.	4.0	3.00-5.00
I am interested in using new digital technologies for myself.	5.0	3.25-5.00
I am curious about the further development of digital technologies in the future.	5.0	4.00-6.00
**Satisfaction**		
Overall, I am satisfied that ChatGPT has been addressed in this course.	5.5	4.25-6.00
Overall, I am satisfied with the topics that ChatGPT has been covered in this course.	5.0	4.00-6.00
The use of ChatGPT has made the corresponding teaching units more interesting.	5.0	4.25-6.00
The integration of ChatGPT in the corresponding teaching units has been implemented well.	5.0	5.00-6.00
**Progress**		
Using ChatGPT has increased my understanding the the respective content regarding placebo and nocebo conveyed.	4.0	3.00-5.00
By using practical examples it has become clearer to me how to use ChatGPT.	5.0	4.00-5.75
By using practical examples it has become clearer to me which possibilities the use of ChatGPT can offer in my current student or later professional everyday life.	5.0	4.00-5.00
On the basis of practical examples it has become clearer to me which limits the use of ChatGPT can have in my current student or later professional everyday life	5.0	4.00-6.00
**Application**		
The application of text-generating AI tools taught using the example of ChatGPT will be useful to me in my everyday student life.	4.0	3.25-5.00
The application of text-generating AI tools taught using the example of ChatGPT will be useful to me in my future professional life.	4.0	3.25-5.00
It became clear to me that a critical evaluation of the generated texts is necessary when using ChatGPT.	5.0	5.00-6.00
The learning units have raised or increased my interest in ChatGPT.	5.0	4.00-6.00

Each item was evaluated on a scale ranging from 1 (no agreement) to 6 (full agreement).

Supplementary Material II: Teaching unit “Expectation”

Questionnaire

(Barth J, Kern A, Lüthi S, Witt CM. Assessment of patients’ expectations: development and validation of the Expectation for Treatment Scale (ETS) BMJ Open 2019;9(6):e026712. doi: 10.1136/bmjopen-2018-026712.)

Please indicate to what extent the individual statements apply to you personally. Please note that there are no right and wrong answers here. We are only interested in your personal opinion

- Item 1: I expect the acupuncture will help me to cope with my complaints.
- Item 2: I expect the acupuncture will make my complaints disappear.
- Item 3: I expect the acupuncture will improve my energy.
- Item 4: I expect the acupuncture will improve my physical performance.
- Item 5: I expect that after the acupuncture, my complaints will be considerably better.

Video content (transcript)

Expert opinion

In 2018, we published a patient-level data meta-analysis on acupuncture for headaches in the Journal of Pain, encompassing nine studies, some of which gained considerable attention in reputable journals like JAMA and the British Medical Journal. The meta-analysis involved over 4000 patients, and the findings are indeed intriguing. When comparing acupuncture to sham acupuncture, where superficial pricking occurs on non-acupuncture points, a statistically significant difference emerges. However, it is crucial to note that the observed effect is relatively small. In essence, the choice of acupuncture points does matter, but the impact is not substantial. Now, if you compare it to standard care, acupuncture yields compelling results. The disparity is not only statistically significant, indicating a non-random effect, but it also holds clinical relevance. This implies that acupuncture demonstrates clinically relevant superiority over standard care.

Clinician opinion

In my medical practice spanning around 15 years, acupuncture has been a consistent component of my consultations. A considerable number of patients suffering from headaches have experienced significant benefits from acupuncture treatments. Typically, we structure these treatments into ten sessions, each spaced a week apart.

The selection of acupuncture points is highly personalized, with the location of the headache serving as a primary consideration. It is important to note that many patients report improvements after just a few initial treatments. For instance, individuals presenting with mild headache symptoms may leave the consultation with a substantial reduction in symptoms. While acupuncture may not completely eliminate the headache, there is a discernible decrease in the frequency or severity of symptoms. It acts as a mitigating factor rather than an absolute remedy.

Patient experience

I suffered from headaches for over 10 years, several times a month, sometimes so severe that my everyday life was very restricted. Sometimes I was even unable to work. I tried prophylactic medication, but they did not help and I did not tolerate it well. I took painkillers for acute pain, but they did not help enough either. Then a friend told me about acupuncture and recommended that I try it. I had a total of 10 sessions, and at the beginning, I didn't really notice any improvement. However, after about four sessions, the headaches were much better. Since then, I've been largely headache-free. Even if my headaches occasionally resurface, the painkillers effectively manage them, allowing me to maintain my daily routine without disruption. Acupuncture has really been an eye-opener for me. Should the headaches intensify again, I would not hesitate to undergo acupuncture treatment once more.

Information from the Internet

Acupuncture is a treatment method in which thin needles are placed in specific areas of the body to relieve pain. Studies have shown that acupuncture can help with tension headaches by reducing the frequency and intensity of headaches. The treatment can also help improve quality of life by increasing well-being and sleep quality.

Acupuncture is a very safe treatment method when performed by an experienced and qualified acupuncturist. Possible side effects are usually mild and temporary, such as slight bruising or pain at the injection site.

The mode of action of acupuncture is not fully understood, but it is believed to work by releasing the body's own painkillers and improving blood flow to the tissues.

If you suffer from tension headaches, acupuncture may be an effective treatment option for you. However, it is important to consult a qualified acupuncturist to ensure that the treatment is safe and effective.

Supplementary Material III: Teaching unit “Information for patients”

Information for patients generated with ChatGPT 3.5

Prompts and texts generated with ChatGPT were in German and translated to English for the publication.

Basic prompt

Can you please write a patient information about the effectiveness of acupuncture for tension headaches. The text should be no longer than 180 words.

Text generated with ChatGPT

Acupuncture is a traditional Chinese medicine in which fine needles are inserted into specific points on the body to relieve pain. Studies have shown that acupuncture can be effective for tension headaches. A meta-analysis of 20 randomized controlled trials found that acupuncture can significantly reduce the frequency of headaches. In addition, acupuncture may also improve pain intensity and quality of life. Although the exact mechanisms of action of acupuncture for tension headaches are not fully understood, it is believed to relieve pain and balance the nervous system through the release of endorphins and other neurotransmitters. If you suffer from tension headaches, acupuncture may be an effective treatment option. However, it is important that you speak with a qualified acupuncturist to find the best treatment for you.

Extended prompt

Can you please change this text so that it is generally understandable for patients? Please formulate statements about effects and side effects in the sense of positive framing. Also make sure that patient safety is guaranteed. The text should be no longer than 180 words.

Text generated with ChatGPT

Acupuncture is a treatment method in which thin needles are placed in specific areas of the body to relieve pain. Studies have shown that acupuncture can help with tension headaches by reducing the frequency and intensity of headaches. The treatment can also help improve quality of life by increasing well-being and sleep quality.

Acupuncture is a very safe treatment method when performed by an experienced and qualified acupuncturist. Possible side effects are usually mild and temporary, such as slight bruising or pain at the injection site.

The mode of action of acupuncture is not fully understood, but it is believed to work by releasing the body's own painkillers and improving blood flow to the tissues.

If you suffer from tension headaches, acupuncture may be an effective treatment option for you. However, it is important to consult a qualified acupuncturist to ensure that the treatment is safe and effective.

Screenshots from interactive whiteboard discussion

Students were instructed to review the text in nine subgroups regarding three criteria comprehensibility (lay language), patient safety and correct application of the communication method (positive framing). Each subgroup reviewed the same text. Review decision of each group was documented by placing coloured dots in the respective sections for each criterium. Additionally, students were asked to label problematic content in the texts by using coloured markers (Table 1).

After basic prompt

(Dots indicate judgments of groups for the three criteria; labeled text indicate unclear, ambiguous and critical terms as identified by the students.)

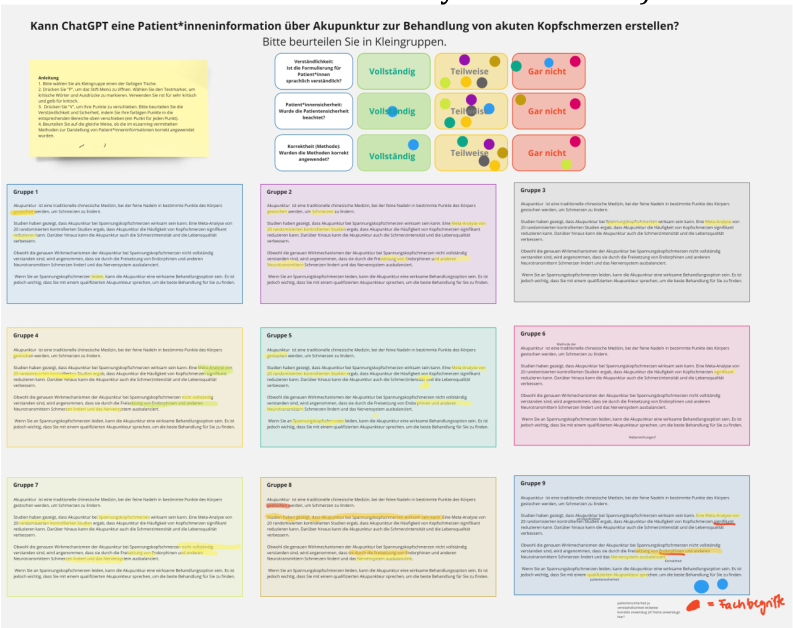

Table 1: Terms labeled as problematic by students group after basic prompt

German	English	N (Group decision counts)
gestochen	pricked	5
Freisetzung von Endorphinen	release of endorphins	5
Neurotransmitter	neurotransmitters	3
Spannungskopfschmerzen	tension headaches	4
Signifikant	significant	3
Meta-Analyse	meta-analysis	5
Nervensystem	nervous system	4
randomisierte kontrollierten Studien	randomized controlled trials	4
Schmerzen	pain	1
leiden	suffer	1
qualifizierte Akupunkteur	qualified acupuncturist	1
Reduzieren	reduce	1

After extended prompt

(Dots indicate judgments of groups for the three criteria; labeled text indicate unclear, ambiguous and critical terms as identified by the students.)

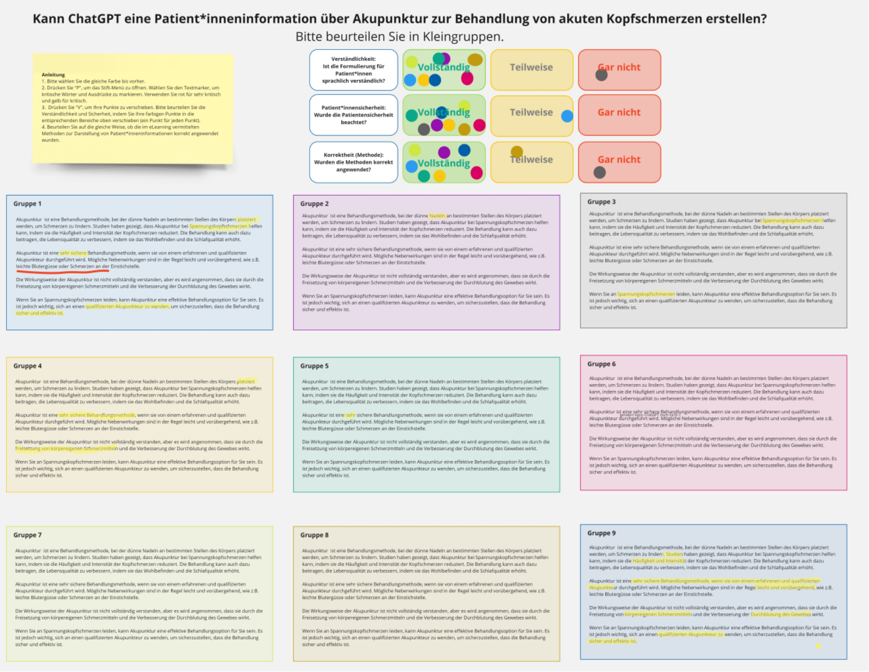

Table 2: Terms labeled as problematic by students group after extended prompt

German	English	N (Group decision counts)
Spannungskopfschmerzen	tension headaches	3
qualifizierter Akupunkteur	qualified acupuncturist	3
sehr sichere Behandlungsmethode	very safe treatment method	3
sicher und effektiv	safe and effective	2
Freisetzung von körpereigenen Schmerzmitteln	release of the body's own painkillers	2
platziert	placed	2
leichte Blutergüsse oder Schmerzen	slight bruising or pain	1
Nadeln	needles	1
Studien	studies	1
Häufigkeit und Intensität	frequency and intensity	1
leicht und vorübergehend	light and temporary	1
Durchblutung des Gewebes	blood circulation of the tissue	1
